# Evaluating the Guideline Enhancement Tool (GET): an innovative clinical training tool to enhance the use of hypertension guidelines in general practice

**DOI:** 10.1186/s12909-014-0273-2

**Published:** 2014-12-30

**Authors:** Chinthaka Balasooriya, Joel Rhee, Boaz Shulruf, Rosa Canalese, Nicholas Zwar

**Affiliations:** School of Public Health & Community Medicine, UNSW Medicine, University of New South Wales, Sydney, Australia; UNSW Medicine, University of New South Wales, Sydney, Australia; GP Synergy and School of Medicine Sydney, University of Notre Dame, Sydney, Australia

**Keywords:** Clinical decision making, Clinical reasoning, Clinical practice guidelines, Guideline Enhancement Tool (GET), General practice training, Clinical education, Hypertension

## Abstract

**Background:**

This project aims to evaluate the effectiveness of an innovative educational intervention in enhancing clinical decision making related to the management of hypertension in general practice. The relatively low level of uptake of clinical practice guidelines by clinicians is widely recognised as a problem that impacts on clinical outcomes. This project addresses this problem with a focus on hypertension guidelines. Hypertension is the most frequently managed problem in general practice but evidence suggests that management of Hypertension in general practice is sub-optimal.

**Methods/design:**

This study will explore the effectiveness of an educational intervention named the ‘Guideline Enhancement Tool (GET)’. The intervention is designed to guide clinicians through a systematic process of considering key decision points related to the management of hypertension and provides a mechanism for clinicians to engage with the hypertension clinical guidelines.

The intervention will be administered within the Australian General Practice Training program, via one of the regional training providers. Two cohorts of trainees will participate as the intervention and delayed intervention groups.

This process is expected to improve clinicians’ engagement with the hypertension guidelines in particular, and enhance their clinical reasoning abilities in general. The effectiveness of the intervention in improving clinical reasoning will be evaluated using the ‘Script Concordance Test’.

**Discussion:**

The study design presented in this protocol aims to achieve two major outcomes. Firstly, the trial and evaluation of the educational intervention can lead to the development of a validated clinical education strategy that can be used in GP training to enhance the decision-making processes related to the management of hypertension. This has the potential to be adapted to other clinical conditions and training programs and can benefit clinicians in their clinical decision-making. Secondly, the study explores features that influence the effective use of clinical practice guidelines. The study thus addresses a significant problem in clinical education.

## Background

The relatively low levels of uptake of clinical practice guidelines is widely recognised in the literature as a practical problem impacting on clinical outcomes [1,2]. This study aims to evaluate the impact of a clinical education intervention designed to enhance the use of clinical practice guidelines and develop clinicians’ clinical reasoning abilities. The study focusses on hypertension guidelines and their use within general practice in Australia.

Hypertension is an important health problem in the Australian general practice setting. Data from the Australian Diabetes, Obesity and Lifestyle (AusDiab) study showed that 28.6% of adults aged 25 years or over had hypertension [3]. The data collected from the Bettering the Evaluation and Care of Health (BEACH) program between January 2009 and 2010 showed hypertension to be the most frequently managed problem in general practice, accounting for 5.7% of all managed problems [4].

Despite the high prevalence and importance of hypertension in the GP setting, there is evidence of a significant deficit in the treatment of hypertension. Data from the AusDiab study showed that only 45.8% of patients with hypertension are being treated [3]. A retrospective study that examined the care provided to 1154 adult Australians showed that of the 4700 clinical encounters involving the management of hypertension, patients received appropriate care only 72% of the time [5].

The reasons for this evidence-practice gap are multi-factorial: while drug-related and patient related factors play a role, health professional-related factors are very important [6,7]. The latter includes difficulties faced by GPs in making management decisions that accommodate patient differences and preferences [8].

This issue was further highlighted in a recent study involving focus groups of Australian GPs that found that the desire to provide comprehensive, holistic care that incorporates patient concerns and context into management decisions was an important factor that affected the management of hypertensive patients [6]. There is therefore a clear need to recognise and respond to the contextual factors that influence clinical decision-making, and to consider these contextual factors in developing and implementing clinical guidelines.

The proposed project aims to address this need through an innovative clinical educational intervention and evaluate its feasibility and effectiveness. The intervention is named the ‘Guideline Enhancement Tool’ (GET) and is designed to:

1. Promote effective use of hypertension guidelines by General Practitioners.
2. Develop clinicians’ decision-making processes related to the management of hypertension.
3. Enable the consideration of contextual factors that influence clinical decisions, and encourage participants to make recommendations for tailoring the guidelines to better suit General Practice contexts.

The design of the GET was informed by previous research [9-11], which highlighted the factors that influence the incorporation of updated evidence to practice. The influences were explored from the perspectives of translational research, and can be framed within the theoretical bases of clinical reasoning [12], evidence-based-medicine [13], and the theory of reasoned action [14]. The clinical education strategy facilitates clinicians’ cognitive engagement with these influences to achieve more effective uptake of guidelines.

## Methods and design

### Study design

This is a non-randomised, delayed intervention study design. The study is not randomised since the educational intervention will be administered in groups and randomising groups will require unrealistic sample size. However, using repeated measures and a delayed intervention design provides an optimal solution without compromising the study quality. Such a design is preferable for evaluation of the educational intervention since all participants receive the intervention and the design enables measure of progress across and within groups, including control for possible (although unlikely) differences between groups at the starting time.

Clinicians participating in this study will be recruited from a population of general practice registrars who are undergoing GPTerm1 or GPTerm2 stages of training at GP Synergy (a major provider of general practice training in Australia), at two training sites. Participants from site A will be the ‘early intervention group’ while the group at site B will be the ‘delayed intervention group’. The two interventions will be identical and will differ only by the time of implementation. Participants will undergo evaluations of clinical reasoning (based on the Script Concordance Test (SCT) [15] upon commencement of project (Time Point 1: before the early intervention), immediately after the early intervention and before the delayed intervention starts (Time Point 2), and at the end of the delayed intervention (Time Point 3).

The impact of the intervention will be measured by comparing the progress each group makes (changes in SCT scores) over each intervention period.

### Sample size

Two systematic reviews suggest that the effect size on SCT between resident doctors and expert doctors ranged 0–3.2; typically around 1 and the effect of educational interventions such as Educational meetings/interactive educational sessions was d > 1 [16,17]. Based on those studies, we expect that the intervention will yield an effect size of d ≥ 1.0 measured by the differences in the SCT results between the intervention and the control groups between Time Point 1 and Time Point 2. A similar increase is expected for the delayed intervention group between Time Point 2 and Time Point 3. Therefore, a sample size of 34 clinicians (17 intervention & 17 control) will have 80% power to detect an effect size of d = 1 (one sd.) with an alpha of 0.05 (two sides) [18].

### The educational intervention

The GET is a clinical educational instrument that has been developed by a team at UNSW Medicine, Australia. It is structured to facilitate a two stage process of engagement. Each stage (requiring approx. 30–45 mins) is designed to fit within existing workshop style GP training sessions. The two stages are based on two instruments (A&B) that have been designed to guide clinicians’ engagement with hypertension guidelines and the key decision points in managing hypertension. The GET will be supplemented by a final panel discussion with experts (experienced general practitioners and a cardiologist) which will enable participants to discuss any concerns and clarify any issues that arises out of their engagement with the GET. This discussion, drawing on participant experiences of the usefulness of the guidelines, also aims to develop recommendations to enhance the usability of the existing guidelines.

The first stage instrument (A) is based on a straightforward hypertension patient presentation, devoid of any complicating contextual factors. This stage provides a framework for clinicians to critically evaluate their decisions in relation to such a hypertension presentation. This stage will focus clinicians on the ‘key decision points’ relevant to the ideal management of such an uncomplicated patient presentation, and will facilitate clinicians’ engagement with hypertension guidelines.

The second stage instrument (B) will be based on a typical patient presentation in a general practice setting, including contextual influences related to patients, practice limitations and time pressure considerations. This stage will provide a guide to the key decision points that need to be considered by the clinicians, with a framework to identify and articulate the reasons for their decisions in relation to the key decision points. This process will also provide a mechanism for clinicians to identify specific contextual factors that influence their decisions.

It is envisaged that this two stage process will not only enhance clinician engagement with hypertension guidelines, but will also enhance their clinical decision making skills.

The second stage instrument (B) also encourages clinicians to reflect on contextual issues particular to the management of hypertension in general practice, and allows them to suggest potential modifications to the guidelines. This is an important contribution to the process of continuously improving the quality of clinical practice guidelines, in order to meet conditions or contexts which might have been overlooked or have become outdated.

The final expert panel discussion will provide an opportunity for these suggestions to be discussed with relevant experts and peers, in order to develop evidence-based, contextually appropriate, consensus guidelines. This will address a commonly identified issue that impacts on the uptake of guidelines: the perceived ‘top-down’ nature of guidelines and accompanying interventions to promote their use, and the limited involvement of end-user clinicians in the process of guideline development [17-19].

The GET will be reviewed by senior GPs and will be trialled on GPs and GP trainees (who have progressed beyond the participants’ stage of training). Feedback from this process will be systematically collected and used to inform further refinements if required.

### Measures

The script concordance test (SCT) is a test designed to assess examinees’ organisation of knowledge for application in clinical decisions [15]. The organisation of knowledge is named a script. This written test is based on authentic clinical scenarios that form the basis for test items. The test items are categorised as diagnostic, investigative or treatment options and examinees are required to rate their agreement with the provided response options based on a combination of clinical information that is provided [15]. While it is a relatively new assessment method, the SCT is well established in the medical education literature as a tool with good psychometric properties and good face validity for assessment of clinical decision-making competence. It is expected that the SCT will enable the evaluation of the impact of the GET on developing clinical reasoning skills.

Prior to commencement of the study, the SCT items will be reviewed by the project team that includes experienced GPs and medical educators. An SCT scoring panel of experienced GPs will be established to ensure scoring that is relevant to the GP trainee context.

Participating registrars will also be invited to participate in semi-structured qualitative interviews that will take about 20–30 minutes, to obtain their views on the effectiveness and feasibility of GET.

Written informed consent will be obtained from participants, using two ‘Participant Information & Consent Forms’ approved by the Human Research Ethics Panel of UNSW Australia (Approval ID: 2014-7-26). Informed written consent will thus be obtained for both the survey stage and the interview stage of the study.

### Statistical analysis

The main analyses in this study will employ Analysis of Variance (ANOVA). (a) Factorial ANOVA will be used to compare the two groups at Time Point 1 and identify possible differences on SCT scores across groups; (b) One-way repeated measure ANOVA will be used to measure the impact of the intervention by comparing differences in SCT scores within individuals and across groups between Time Point 1–2 and between Time Point 2–3. This analysis is appropriate as it allows identifying the source for the change in the SCT scores (intervention, initial level of competence or errors [20], and testing the hypothesis that the change in the SCT was made by the intervention. We set up a significance level of p < .05 to demonstrate that the intervention was successful.

### Ethics approval and funding

This study has ethics approval from the Medical and Community Human Research Ethics Advisory Panel of the University of New South Wales (Reference Number: 2014-7-26).

The study is funded by a Vanguard Grant (award ID 100260) from the National Heart Foundation of Australia.

## Discussion

This project aims to evaluate the effectiveness and feasibility of an innovative clinical educational intervention named the Guideline Enhancement Tool (GET). The study will explore the role of the GET in enhancing clinical reasoning in general, and specifically related to the management of hypertension in general practice. The project focusses on the important area of clinician engagement with clinical practice guidelines, and includes a bi-directional format that can lead to the enhancement of the guidelines.

The project is expected to result in three major outcomes:

1. Enhanced engagement of general practitioners with hypertension guidelines
2. A validated clinical education strategy that can be used in GP training to enhance the decision-making processes related to the management of hypertension. This has the potential to be adapted to other clinical conditions and training programs
3. Recommendations for enhancing the feasibility and practicality of hypertension guidelines within general practice contexts

As the clinical education strategy aims to enhance clinicians’ clinical reasoning abilities, it has the potential to benefit clinicians in their clinical decision-making across many areas, beyond the focus of this project. The educational strategy also serves as a model to inform the process of future clinical guideline development.

The study design presented in this protocol explores features that relate to the effective use of clinical practice guidelines. The study thus addresses a significant problem in clinical education that can benefit from exploration from a new perspective.

## Abbreviations

GET: Guideline enhancement tool
GP: General practitioner
SCT: Script concordance test
UNSW: University of New South Wales
